# Rod migration to the occiput after C3–7 instrumentation: A rare case report and literature review

**DOI:** 10.1016/j.ijscr.2024.110425

**Published:** 2024-10-19

**Authors:** Reza Jabbari, Ibrahim Mohammadzadeh, Behnaz Niroomand, Ahmad Jabbari, Mehdi Darekordi, Seyed Ali Mousavinejad

**Affiliations:** Skull Base Research Center, Loghman-Hakim Hospital, Shahid Beheshti University of Medical Sciences, Tehran, Iran

**Keywords:** Posterior cervical stabilization, Rod migration, Cervical canal stenosis, Cervical myelopathy, Neurosurgery

## Abstract

**Introduction and importance:**

Cervical canal stenosis often requires posterior laminectomy with lateral mass (LM) screw/rod fixation for sagittal stability. Although rare, rod migration can pose serious risks, such as penetration into cranial structures, emphasizing the need for vigilant postoperative monitoring and prompt intervention.

**Case presentation:**

A 65-year-old male with no significant prior medical history underwent C3–7 laminectomy with LM screw/rod fixation for cervical canal stenosis. Two months postoperatively, the patient experienced persistent neck pain. Imaging revealed right-sided rod migration into the occipital bone, confirmed by CT scan. Urgent revision surgery was performed to remove the migrated rod, resulting in a successful recovery without further complications during follow-up evaluations.

**Clinical discussion:**

Rod migration is a rare but serious complication of LM screw/rod fixation, influenced by technical factors such as screw placement, angulation, and rod length. Accurate preoperative planning, meticulous surgical technique, and detailed postoperative surveillance are crucial in preventing such occurrences. This case highlights the significance of recognizing potential hardware complications early, facilitated by imaging modalities like CT, to avoid severe neurological outcomes.

**Conclusion:**

This case underscores the necessity of thorough preoperative assessment, precise surgical execution, and rigorous postoperative monitoring in managing cervical spine stabilization surgeries. Improved diagnostic imaging and prompt surgical intervention are key to mitigating risks associated with rod migration, ultimately enhancing patient outcomes.

## Highlights

• The study explores LM screw/rod fixation for cervical canal stenosis, highlighting its role in sagittal stability.
• This infrequent complication demonstrated improvement after the reporting of symptoms and appropriate management.
• The importance of post-surgery follow-up after device placement

## 1 Introduction

Cervical canal stenosis is characterized by the narrowing of the spinal canal in the cervical region, resulting in nerve root compression and subsequent myelopathy. Contributing factors encompass degenerative alterations within the cervical spine, notably spondylosis, disk degeneration, osteophyte formation, ligamentous thickening, and facet hypertrophy [1,2].

For treatment, posterior cervical laminectomy (PCL) is the standard surgical approach. However, to secure sagittal stability in patients with multilevel cervical canal stenosis, fixation of lateral mass (LM) screw/ rod is of benefit. The use of an LM screw/ rod in PCL provides immediate stability to the cervical spine after laminectomy and avoids complications of standalone laminectomy, such as cervical kyphosis [[3], [4], [5]].

Despite advances in techniques and methods, surgical complications remain; in this method, Complications can be categorized into short-term concerns, including infection, hematoma, and neurological deficits, and long-term issues, such as screw or rod fractures [[6], [7], [8], [9]].

However, the few existing cases provide critical insights into this complication. For example, A 61-year-old female experienced a persistent headache due to rod migration six months after cervical spine surgeries. The rod was found to have protruded into the occipital bone [10]. Another case reported was rod migration into the posterior cranial fossa 48 months after C1–4 posterior rod fixation. The authors speculated that the cervical spine's unique anatomical and functional characteristics influenced the migration. This highlights the potential for delayed complications in spinal fixation procedures [7]. Notably, the underlying conditions necessitating surgical intervention, the presenting symptoms, and the time elapsed from the initial surgery varied significantly across the cases reviewed in this article (Table 1).

**Table 1:** Study characteristics of reported cases of cervical rod migration after posterior laminectomy and fusion, since 2010.

First author [Ref.]	Year	Gender (M:male, F:female)/ Age	First surgery indication	First operation method	Symptoms regarding rod migration	Time to migration (month)	Site of rod migration	Possible etiology(s)	Management	Outcome
Chun et al. [1]	2010	M/ 23	Odontoid fracture during traffic accident	C1lma–2p^b^ fixation (Harms method)	Headache Dizziness	20	Right cerebellar hemisphere	–	Refused revision surgery	Persistent severe brain damage and residual sequala of his accident trauma
Plant and Ruff [2]	2010	M/ 13	Forced neck flexion injury during a rugby scrum	C1–2 posterior instrumented fusion and autologous bone grafting for C1–2	Neck pain (on extension)	36	Right cerebellar hemisphere	Trauma	Revision surgery	–
Chalouhi et al. [3]	2013	F/ 82	–	Anterior–posterior occipitocervical fusion	Right-sided weakness Headache	120	Left cerebellar hemisphere	–	Follow-up imaging	Stable size of hemorrhage
Nakao et al. [4]	2014	M/ 70	AAI^c^ due to RA^c^	C1lm-2p fixation (Harms method)	Headache (on extension)	3	Occipital bone	Angle of screw insertion Length of rod protrusion	Revision surgery	Resolution of symptoms
Kiran et al. [5]	2016	M/ 55	Ossification of posterior longitudinal ligament	Posterior cervical decompression and lm screw fixation	LOC^e^	18	Left cerebellar hemisphere	–	Intubation at ED^f^^s^ Emergency revision surgery	Death at day 8th post-op
Miyaoka et al. [6]	2017	F/ 81	Sensory disturbance and motor weakness in upper extremities due to AAI	C1lm-C2p fixation (Harms method)	Severe headache Neck Pain in protrusion position Vomiting	1	Occipital bone	Length of rod protrusion Placing of patient's head in the protrusion position after surgery	Revision surgery	Resolution of symptoms
Hammoud et al. [7]	2021	F/ 56	Odontoid fracture during traffic accident	C1–2 posterior fusion using laminar hooks	No neurological symptoms	1.5	Posterior cranial fossa	Weakly locked rod	Revision surgery	Patient remained symptom-free
Mahtabfar et al. [8]	2021	F/ 70	AAI due to RA^d^	C1–2 fusion later followed by revision cervicothoracic fusion due to degeneration	Headache Nausea Vomiting	180	Posterior cranial fossa	Length of rod protrusion Kyphotic and sagittal imbalance	Revision surgery	Resolution of symptoms
Ezzat et al. [9]	2022	F/ 67	Cervical canal stenosis	C3–6 lm fixation	Headache Right-sided facial palsy Complete right ophthalmoplegia	24	Posterior cranial fossa	Trauma	Revision surgery	Marked resolution of headache with persistent ophthalmoplegia and facial palsy
Basankin et al. [10]	2023	M^7^/ 25	C2–3 fracture during traffic accident	C1–4 posterior fixation	Severe pain in the cervical spine and occipital region Visual impairment Nausea	48	Posterior cranial fossa	Anatomical and functional features of the cervical spine	Revision surgery Referral to ophthalmologist	Resolution of symptoms
Hirata et al. [11]	2024	F^8^/ 61	Destructive spondyloarthropathy (DSA)	Anterior cervical corpectomy with C5-T1 fusion followed by C2-T2 posterior fusion and fixation of C2 laminar hook	Headache	6	Occipital bone	Length of rod protrusion Background condition (DSA)	Revision surgery	Resolution of symptoms

a Lateral mass.

b Pedicle.

c Atlantoaxial instability.

d Rheumatoid arthritis.

e Loss of consciousness.

f Emergency department.

Published case reports document various complications and symptoms associated with rod displacement, including migration from cervical spine fixation sites to the occipital region, penetrating cranial structures as tabulated in Table 1. These cases highlight the seriousness of such complications and emphasize the need for careful monitoring and timely action.

This case report presents an exceptional and rare case of rod migration to the occiput region via a subdural route following PCL and LM screw/ rod fixation of C3–7 vertebrae. Moreover, related case report studies were reviewed to help readers understand the various complications of rod migration in posterior stabilization surgeries.

This case report has been reported in line with the SCARE criteria [11].

## 2 Case presentation

**Fig. 1:**
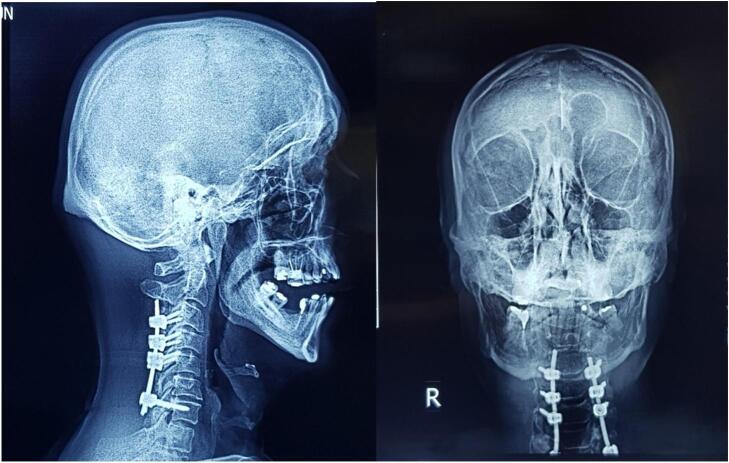
Sagittal (A) and coronal (B) X-ray radiographs of the patient's cervical spine following posterior cervical laminectomy and lateral mass screw/rod fixation from C3 to C7. The images show the alignment and positioning of the surgical hardware, providing an overview of the postoperative spinal stabilization.

The patient was a 65-year-old male with no significant past medical history who initially presented with symptomatic cervical stenosis characterized by chronic neuropathic pain, paresthesia in the upper limbs (predominantly affecting the right arm), and reduced grip strength in the hand (Fig. 1). Given the severity of the symptoms and radiological evidence of significant cervical stenosis, surgical intervention was deemed necessary.

**Fig. 2:**
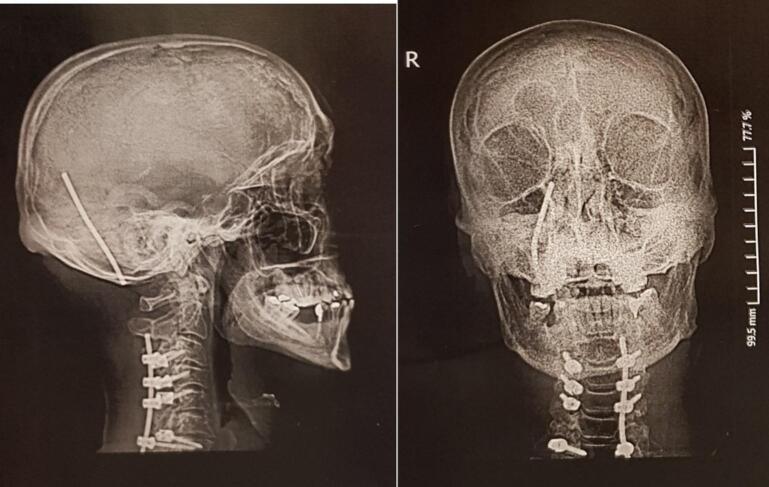
Coronal (A) and sagittal (B) X-ray radiographs of the patient's cervical spine four months postoperatively, showing right-sided rod migration into the occipital region. The images illustrate the displacement of the hardware, highlighting the need for urgent revision surgery.

Considering that the patient underwent an operation at another center, where a laminectomy was performed from the C3 to C7 vertebrae, and a fusion was achieved using lateral mass screws from C3 to C6 and a pedicle screw at C7, all secured with a 13 cm rod. Two months postoperatively, the patient began to experience persistent neck pain. Despite receiving symptomatic treatment, the symptoms persisted for another two months, leading the patient to seek further evaluation at our center. Upon presentation, the patient exhibited tenderness in the cervical region. Neurological examination revealed intact motor function and sensation, though the patient continued to report neck pain and discomfort (Fig. 2).

**Fig. 3:**
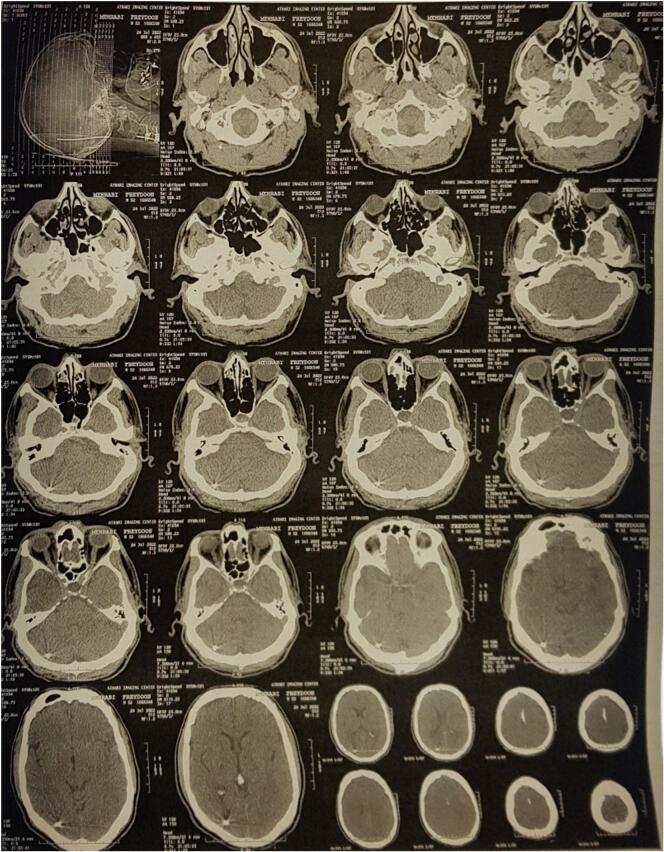
Axial CT scans of the brain, obtained four months postoperatively, showing the precise localization of the dislocated rod. The images highlight the migration path of the rod into the cranial cavity, with arrows indicating the rod's position relative to surrounding structures.

**Fig. 4:**
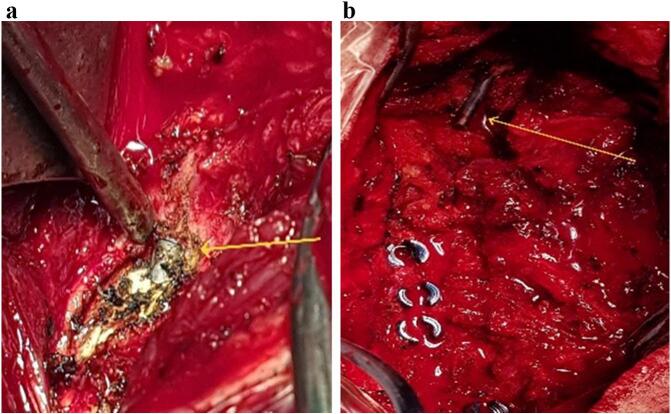
(A & B). Intraoperative images showing the dislocated rod during revision surgery. The arrows indicate the migrated hardware, which penetrated into the subdural space. The images illustrate the rod's position relative to the surrounding tissues, highlighting the complexity of its retrieval and the surgical approach required to correct the displacement.

Cervical plain radiographs showed a dislocation of the right-sided rod, with extension into the skull (Fig. 2). A non-contrast computed tomography (CT) scan was conducted to more accurately identify the anatomical position, revealing that the rod had penetrated the right squamous part of the occipital bone and migrated into the posterior fossa, passing through the tentorium and sliding into the subdural space of the supratentorial region, located behind the occipital lobe. Fortunately, there were no other complications, such as subdural hematoma or leakage and accumulation of cerebrospinal fluid (Fig. 3). Given the critical findings, the patient was urgently taken to the operating room for corrective surgery. The previous surgical site was reopened, and careful muscle dissection was performed on the right side to access the lateral mass screws and the protruding rod. Intraoperative findings confirmed that the rod had migrated into the skull, penetrating the subdural space (Fig. 4a & b). The rod was carefully extracted (Supplementary material, video 1), repositioned, and securely reattached to restore proper alignment and stability of the cervical spine.

The patient was admitted to the surgical intensive care unit postoperatively for close monitoring, remained stable, and discharged after two days. Follow-up evaluations on postoperative days 10, 30, and 60 revealed no complications and reported resolution of the neck pain, and imaging confirmed the maintained alignment and stability of the cervical spine.

## 3 Discussion

This case describes a rare case of rod migration into the occiput that occurred after C3–7 laminectomy and lateral mass (LM) screw/rod fixation. In this context, the findings emphasize the importance of monitoring during the postoperative period and the physician's awareness of the potential risks to avoid the further unfavourable course of hardware-related complications.

Two main approaches to correcting cervical canal stenosis are anterior cervical decompression and fusion (ACDF) and posterior decompression. Posterior decompression can be performed by laminoplasty, flavectomy, laminotomy, foraminotomy, and laminectomy [1]. Before the introduction of anterior approaches, laminectomy was the most frequently used surgical method for cervical spondylotic myelopathy (CSM). However, standalone laminectomy faced many challenges, including post-laminectomy kyphosis, which has been addressed by implementing a posterior cervical fusion simultaneously with decompression [2]. Instrumented posterior cervical fusion provides safe stabilization, does not interfere with decompression, and permits early patient mobilization [2]. Pedicle/LM screw-rod fusion after laminectomy remains a valuable tool for cervical decompression in selected cases of multilevel CSM (≥3-multilevelase) with neutral or lordotic cervical alignment or subclinical instability to prevent post-laminectomy instability and kyphosis [1,3].

In a systematic review of posterior cervical fusion and decompression surgery outcomes, the reported complications of LM screw/ rod system for fusions of C3–7 levels mainly included C5 palsy, axial pain, wound infection, and CSF leakage [4]. Rod migration to occiput is a rare complication of LM screw/ rod fixation, with only 10 cases reported in the literature since 2010, all of which have undergone C1–2 or occipitocervical fixation, except for one with C3-C6LM screw/ rod fixation. Surgery as soon as possible as a quick response against facing such complications in follow-ups contrasts with cases like those reported by Acaroğlu et al., where delayed identification of rod migration led to severe patient outcomes, emphasizing the need for regular postoperative imaging and proactive management strategies [12].

In our review of the literature, among 10 cases, seven were operated on due to chronic diseases, and four were after acute fractures during traffic accidents. The most frequent procedure included C1–2 fusion followed by occipitocervical fusion, C1–4 posterior fixation, and anterior C5-T1 with subsequent posterior C2-T2 fusion. The longest and shortest time gap to the emergence of migration's symptoms were 180 and one month(s), respectively. Several etiologies are described for rod migration in the literature, including; trauma to the neck, degenerative bone diseases, and intraoperative technical errors such as inappropriate rod protrusion, weak rod locking, and screw fixation angle (Table 1). Anatomical and biomechanical factors of the cervical spine, such as rod length, kyphotic deformity, sagittal imbalance, inadequate initial fixation, improper alignment, and dynamic cervical spine movements post-surgery, can contribute to complications like rod migration. Mahtabfar et al. described a case where these factors influenced rod migration, highlighting the significance of early detection and regular imaging to prevent cervical spine hardware complications. Comprehensive preoperative planning, including flexion-extension X-rays to identify potential alignment issues, is essential to ensure correct cervical alignment and sagittal balance during surgery. Proper surgical techniques, such as 360-degree fixation or occipital-cervical fusion, are critical to minimizing rod migration risks. Additionally, maintaining rod protrusion below 2 mm further reduces hardware movement risks [6].

Other patient-specific factors, such as bone resorption, comorbidities like rheumatoid arthritis, and age-related conditions like osteopenia and osteoporosis, can further complicate biomechanical integrity, leading to cranial settling and increased risk of rod and screw failure. Screw length, determined by bone thickness, plays a crucial role in mitigating screw failure, particularly in older patients with thinning bones. Thorough pre- and post-operative assessments of the fused bone are necessary to reduce the risk of rod and screw-related complications. This underscores the need for meticulous preoperative planning and selecting an appropriate surgical approach [5]. One pathophysiology explained by Basankin et al. suggests biomechanical failure can happen in case of posterior screw fixation in a functioning spine, particularly in the long-term period. Preserved disk mobility while the facet joints are blocked and their mobility is limited leads to a chronic increased load on the components of the rigid fixation system and increases the risk of developing mechanical complications such as a fracture of the rod/screw, rod migration, or screw loosening [7]. It is also hypothesized that long-term complications are likely to result from hardware failure rather than a surgeon's error because it would have manifested earlier.

Post-operative radiograph is the most frequent approach for follow-up of the patients after rod/ screw fixation. However, a consensus on the follow-up period has yet to be reached. Most of the authors suggest two years post-operatively [9,[13], [14], [15]].

In conclusion, this case emphasizes the need for greater awareness of rod migration as a severe, albeit rare, complication of PCL and LM screw/rod fixation. The rod migration observed in this case may be influenced by several subjective technical factors, including intraoperative decisions on screw placement, angulation, rod length, and the secure fit of hardware, as well as the handling of soft tissues. Suboptimal positioning or fixation can increase mechanical stress, contributing to hardware migration. The surgeon's experience and meticulous attention to detail during fixation are critical to reducing complications. This case underscores the importance of thorough preoperative evaluation, precise surgical techniques, careful stabilization, regular and accurate postoperative follow-up, and early intervention at diagnosis. Incorporating these lessons into clinical practice can improve patient outcomes, reduce complications, and enhance surgical decision-making in similar cases.

## 4 Conclusion

the case emphasizes the necessity of vigilant surveillance post-cervical spine stabilization surgeries, particularly in addressing rare complications like rod displacement. Prompt intervention proved crucial in managing the presented case, leading to a successful outcome without further complications. The discussion underscores the advantages of posterior decompression techniques and LM screw-rod fusion for stability, albeit with associated risks. Comprehensive preoperative assessments and meticulous postoperative monitoring are essential to mitigate hardware complications and ensure favorable patient outcomes. This highlights the critical role of careful assessment and surveillance in navigating the complexities of cervical spine surgery.

The following is the supplementary data related to this article.

Video S1 Video showing the removal of a migrated rod from the occiput after C3–7 instrumentation. Video S1

## Author contribution

Ibrahim Mohammadzadeh (IM), Behnaz Niroomand (BN), and Seyed Ali Mousavinejad (SAM) conceptualized the manuscript, reviewed the literature, and wrote the original draft. IM, Ahmad Jabbari (AJ), and BN designed the study. SAM and Reza Jabbari (RJ) performed the surgical procedure. Mehdi Darekordi (MD) edited the manuscript. IM and SAM supervised the entire study process. All authors read and approved the final manuscript.

## Consent

The authors of this manuscript confirm that written informed consent has been obtained from the patient for the publication of the case report and any accompanying images. The consent process involved providing the patient with clear and understandable information about the study's purpose, procedures, potential risks, and benefits. The patient had the opportunity to ask questions and have their concerns addressed before voluntarily agreeing to participate in the publication of their case. A copy of the written consent form is available for review by the editor-in-chief of this journal upon request, ensuring transparency and accountability in the consent process.

## Ethical approval

Ethical approval is not required since our patient's treatment was based on approved options, and it was not found to be controversial, according to the Ethics Committee of our institution (Iran National Committee for Ethics in Biomedical Research).

## Funding

There is no funding source.

## Guarantor

Seyed Ali Mousavinejad.

## Registration of research studies

Not applicable.

## Declaration of competing interest

Authors declared no personal or financial conflicts of interest.
